# Metabolic Acidosis and Strong Ion Gap in Critically Ill Patients with Acute Kidney Injury

**DOI:** 10.1155/2014/819528

**Published:** 2014-08-05

**Authors:** Cai-Mei Zheng, Wen-Chih Liu, Jing-Quan Zheng, Min-Tser Liao, Wen-Ya Ma, Kuo-Chin Hung, Chien-Lin Lu, Chia-Chao Wu, Kuo-Cheng Lu

**Affiliations:** ^1^Division of Nephrology, Department of Internal Medicine, Shuang Ho Hospital, Taipei Medical University, Taipei, Taiwan; ^2^Graduate Institute of Clinical Medicine, College of Medicine, Taipei Medical University, Taipei, Taiwan; ^3^Division of Nephrology, Department of Internal Medicine, Yonghe Cardinal Tien Hospital, New Taipei City, Taiwan; ^4^Department of Internal Medicine, Cardinal Tien Hospital, School of Medicine, Fu-Jen Catholic University, New Taipei, Taiwan; ^5^Department of Pediatrics, Taoyuan Armed Forces General Hospital, Taoyuan 325, Taiwan; ^6^Division of Nephrology, Department of Medicine, Shin Kong Wu Ho-Su Memorial Hospital, Taipei, Taiwan; ^7^Division of Nephrology, Department of Medicine, Tri-Service General Hospital, National Defense Medical Center, Taipei, Taiwan

## Abstract

*Purpose*. To determine the influence of physicochemical parameters on survival in metabolic acidosis (MA) and acute kidney injury (AKI) patients. *Materials and Methods*. Seventy-eight MA patients were collected and assigned to AKI or non-AKI group. We analyzed the physiochemical parameters on survival at 24 h, 72 h, 1 week, 1 month, and 3 months after AKI. *Results*. Mortality rate was higher in the AKI group. AKI group had higher anion gap (AG), strong ion gap (SIG), and apparent strong ion difference (SIDa) values than non-AKI group. SIG value was higher in the AKI survivors than nonsurvivors and this value was correlated serum creatinine, phosphate, albumin, and chloride levels. SIG and serum albumin are negatively correlated with Acute Physiology and Chronic Health Evaluation IV scores. AG was associated with mortality at 1 and 3 months post-AKI, whereas SIG value was associated with mortality at 24 h, 72 h, 1 week, 1 month, and 3 months post-AKI. *Conclusions*. Whether high or low SIG values correlate with mortality in MA patients with AKI depends on its correlation with serum creatinine, chloride, albumin, and phosphate (P) levels. AG predicts short-term mortality and SIG value predicts both short- and long-term mortality among MA patients with AKI.

## 1. Introduction

Metabolic acidosis is an acid-base disorder of the blood and is an especially challenging condition among patients in intensive care units (ICUs). The acid-base status of a patient is most often determined based on standard base excess (SBE), serum bicarbonate levels, and serum anion gap (AG) [1]. However, electrolyte and protein abnormalities, such as hypoalbuminemia and sepsis, can confound the analysis of such serum biochemical indicators of acid-base disorders [2].

An alternative method of acid-base evaluation has been proposed by Stewart [3] and was later modified by Figge et al. [4–6]. This method is based on the analysis of the strong ion difference (SID), the partial pressure (Pa) of carbon dioxide in the blood, and the total weak acid concentration in the serum. The SID is the difference between the levels of fully dissociated anions and cations in the serum, and the total weak acid concentration is primarily determined by albumin and phosphate, which influence blood pH and the concentration of bicarbonate. The analysis of these serum parameters allows the identification of acid-base disorders in complex clinical situations which might otherwise confound their identification based on AG and bicarbonate level alone and provides insight regarding the underlying pathology [7]. Recent studies have used this method to evaluate metabolic acid-base disorders in critically ill patients under various conditions [8, 9].

Although the physicochemical theory proposed by Stewart has served as the basis for understanding the mechanisms of metabolic acid-base disorders, it remains unclear how the physicochemical factors involved in metabolic acidosis influence mortality in critically ill patients with acute kidney injury (AKI). In our current study, we evaluated the prognosis of ICU patients with metabolic acidosis based on their Simplified Acute Physiology Score (SAPS-II) and their results on the Acute Physiology and Chronic Health Evaluation- (APACHE-) II and IV, and we examined the influence of the apparent strong ion difference (SIDa), strong ion gap (SIG), AG, and corrected anion gap (CAG) on mortality at 24 h, 72 h, one week, one month, and 3 months post-AKI.

## 2. Materials and Methods

### 2.1. Study Population

We performed a prospective observational cohort study in the nephrology intensive care unit (ICU) of an urban hospital from February 18 to August 17, 2012. Our study was approved by the local ethics committee. The requirement for informed consent was waived because our study did not interfere with routine clinical measurements. Our study cohort included 78 adult, critically ill patients with metabolic acidosis, defined as blood pH less than 7.35 and an SBE of −5 or less, that was present at the time of ICU admission or developed during their hospitalization. The APACHE-II and -IV were administered, and the SAPS-II were recorded for each patient. Patients were grouped into the AKI and non-AKI groups. Data for the non-AKI group were compared with those of the AKI group. The data for the survivors (*n* = 20) and nonsurvivors (*n* = 20) in the AKI group were compared to examine the relationship between the physicochemical parameters and mortality at 24 h, 72 h, 1 week, 1 month, and 3 months following the onset of AKI. Standard ICU care was provided to each patient to maintain hemodynamic conditions, and renal replacement therapy was administered as needed.

### 2.2. Definition of AKI

We used the RIFLE criteria (risk, injury, failure, loss, and ESRD) to classify the patients according to their creatinine levels [10]. The baseline creatinine level of each patient was defined as the lowest value recorded during the 1-month period immediately preceding their ICU admission. If their preadmission creatinine levels were not available, the Modification of Diet in Renal Disease equation [11] was used to define their baseline creatinine level, based on an estimated lower limit for normal baseline glomerular filtration rate of 75 mL/min. Although some patients have no recorded creatinine prior to AKI, they all have RIFLE-injury and failure status at the time of AKI, which made them unlikely to be misclassified by MDRD equation.

### 2.3. Measurements

All acid-base variables and clinical data were collected from electronic patient charts. Arterial lactate, hemoglobin, and serum levels of albumin, creatinine, and various electrolytes, including Na^+^, K^+^, Ca^2+^, Mg^2+^, Cl^−^, and HPO_4_^2−^, were recorded for each patient. Blood pH and the partial pressures of oxygen and carbon dioxide in the blood (PaO_2_ and PaCO_2_) were measured using a RapidLab Blood Gas Analyzer (Bayer Healthcare, Leverkusen, Germany). The blood bicarbonate level was calculated by using the Siggaard-Andersen formula, which is based on the Henderson-Hasselbalch equation, as pH = 6.1 + log ([HCO_3_^−^]/[CO_2_]), where [CO_2_] = 0.0301 × PaCO_2_. The SIDa, SIDe, SIG, AG, albumin, and albumin corrected CAG were calculated as follows, with electrolyte concentrations expressed in meq/L: SIDa = [Na^+^] + [K^+^] + [Ca^2+^] + [Mg^2+^] − [Cl^−^] − [lactate]; SIDe = 12.2 × PaCO_2_ [(mm Hg)/(10^pH^)] + {[albumin (g/L)] × [0.123 × pH − 0.631] + [HPO_4_^2−^(mmol/L)] × [0.309 × pH − 0.469]}; SIG = SIDa − SIDe; AG = [Na^+^] − [Cl^−^] − [HCO_3_^−^]; and CAG = AG + {(2.5)(4 − [albumin (g/L)]}.

### 2.4. Statistical Analysis

Data are presented as means and standard deviations for continuous variables and as a percentage for categorical variables. The distributions of continuous variables were examined by the Shapiro-Wilk test. Student's* t*-test, Wilcoxon rank-sum test, and chi-square test were used to compare characteristics in those with or without acute kidney injury and in those who survive or not. Pearson's correlation was used to assess the relationship of the parameters with clinical characteristics and mortality. A two-tailed *P* value below 0.05 was considered significant. Statistical analyses were performed using the Statistical Package for the Social Sciences (SPSS/PC; SPSS, Inc., Chicago, IL, USA).

## 3. Results

The characteristics of the AKI and non-AKI patients with metabolic acidosis are summarized in Table 1. The AKI (*n* = 40) and non-AKI (*n* = 38) patients were similar with respect to age (72.5 ± 17.9 versus 67.3 ± 10.4, *P* = 0.12), sex (men/women; 22/18 versus 18/20, resp., *P* = 0.5), blood pH (7.20 ± 0.15 versus 7.22 ± 0.11, *P* = 0.42), SBE (−13.68 ± 6.31 versus −11.86 ± 3.88, *P* = 0.098), serum albumin level (2.95 ± 0.60 versus 2.89 ± 0.0.57, *P* = 0.65), and serum lactate level (67.3 ± 61.7 versus 63.3 ± 42.2, *P* = 0.743). Sepsis was diagnosed in a majority of the patients.

The levels of BUN, Cr, uric acid, and phosphate; the WBC count; mortality; the AG, SIDa, and SIG values; and the APACHE scores of the AKI group were higher than those of the non-AKI group (Table 1). We evaluated the effects of the laboratory parameters on survival in patients with metabolic acidosis who developed AKI (Table 2). The SIDa (37.24 ± 4.78 versus 32.159 ± 8.89, *P* < 0.005), SIG (20.85 ± 6.71 versus 13.35 ± 6.18, *P* < 0.15), AG (23.82 ± 11.31 versus 17.15 ± 5.87, *P* < 0.05), and Cr (7.1 ± 3.7 versus 4.4 ± 2.56, *P* < 0.05) values were all significantly higher among the AKI survivor, compared with those of the nonsurvivors (Table 2).

In the AKI group, the SIG value positively correlated with the serum levels of phosphate, creatinine, and albumin and negatively correlated with the serum level of chloride (Figure 2). The APACHE IV scores negatively correlated with the SIG value (*r* = −0.403, *P* < 0.05) and the serum level of albumin (*r* = −0.345, *P* < 0.05; Table 3). The SIG value and the serum creatinine and albumin levels decreased with increasing APACHE IV scores, whereas the serum chloride level increased. We also found a negative correlation between serum bicarbonate levels with SIG levels (*r* = −0.40, *P* < 0.05), AG (*r* = −0.63, *P* < 0.01), and cAG (*r* = −0.59, *P* < 0.01). cAG and AG are found to be more significantly correlated with bicarbonate than SIG. The results of the Pearson correlational analysis of the relationship between mortality and SIG, AG, and CAG at 24 h, 72 h, 1 week, 1 month, and 3 months post-AKI are shown in Table 4. The AG was strongly associated with mortality at 1 and 3 months after the onset of AKI. However, SIG was more closely associated with mortality at 24 h, 72 h, 1 week, 1 month, and 3 months following the onset of AKI. Thus, SIG was a better predictor of overall mortality than AG or CAG in acidotic patients with AKI.

## 4. Discussion

In our current study, we examined whether blood physicochemical parameters influenced survival in critically ill patients with metabolic acidosis and AKI. In these patients, higher plasma Cr, AG, and plasma SIDa and SIG values were associated with a higher survival rate. The SIG value correlated with serum levels of phosphate, creatinine, albumin, and chloride in the AKI group. We also found that, while the SIG value and the levels of creatinine and albumin decreased, the serum level of chloride increased with increasing APACHE IV scores. The AG was strongly associated with mortality at 1 and 3 months post-AKI. However, the SIG was the strongest predictor of mortality at 24 h, 72 h, 1 week, 1 month, and 3 months post-AKI.

In the AKI and non-AKI groups, blood pH, SBE, albumin, and lactate were not significantly different. Mortality; WBC count; APACHE IV score; BUN; serum levels of Cr, potassium, uric acid, and phosphate; and the AG, SIDa, and SIG values were higher in the AKI group than the non-AKI group (Table 1). Metabolic acidosis is common in AKI patients and is associated with higher mortality. The major renal defense against metabolic acidosis is the net urinary excretion of strong anions, such as chloride, and the retention of strong cations [3]. Metabolic acidosis may be related to an impaired ability to excrete strong anions in the urine [12], resulting in an accumulation of metabolic acids, such as orotic acid, oxalic acid, and kynurenic acid [13]. Although CAG and AG have more significant correlation with bicarbonate than SIG, SIG was also found to be negatively correlated with HCO_3_^−^ levels (*r* = −0.632, *P* < 0.05), since SIG is also affected by other anions, including uremic toxins and phosphate. We found that SIG value was associated with serum phosphate levels in AKI patients (*P* < 0.05). Uric acid was significantly higher in the AKI group than the non-AKI group, which might have contributed to the SIG values of the AKI patients. We also observed significant incidences of hypochloremia and hypocalcemia in the AKI group. Hypochloremia and high SIDa in the AKI group might have been the result of less liberal fluid policy in that group, which is consistent with the findings of a recent study [12].

**Figure 1:**
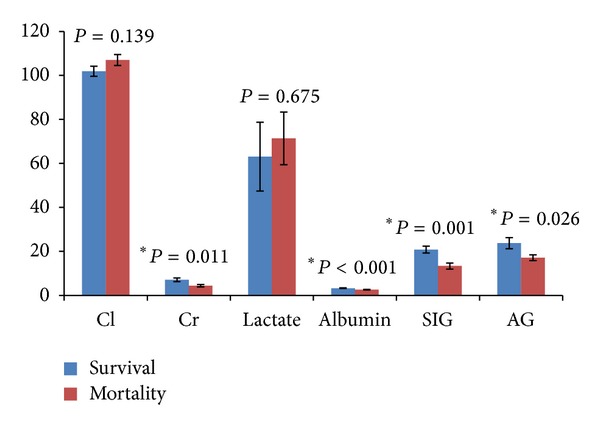
Survival and mortality among AKI patients according to their blood physiochemical parameters.

**Figure 2:**
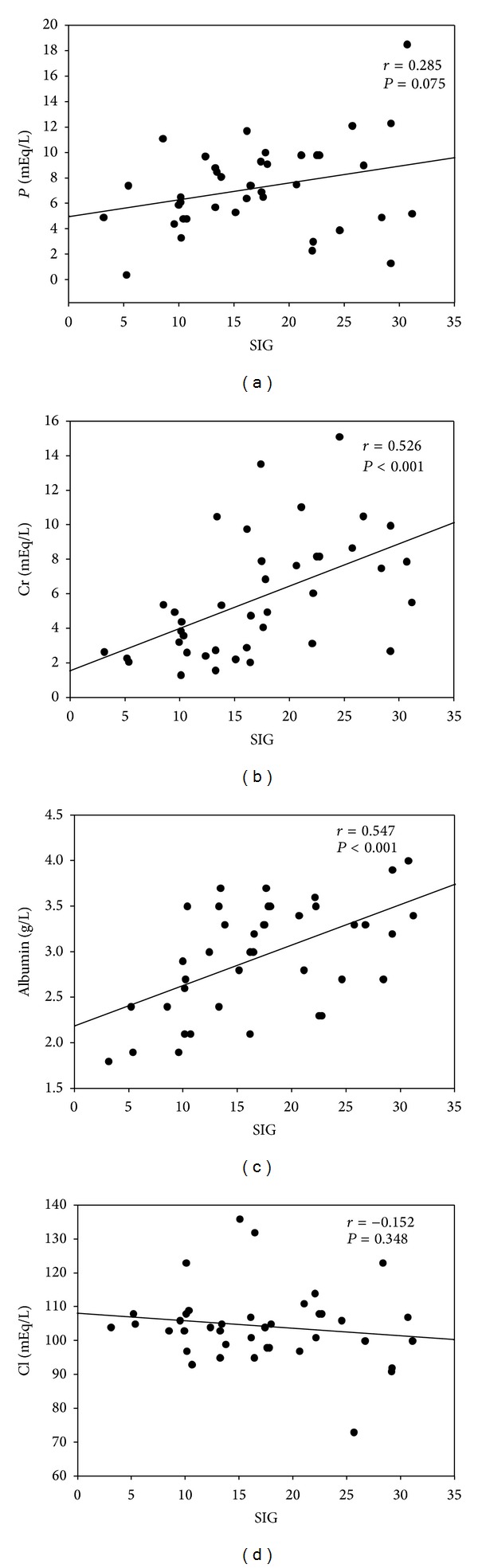
Association of plasma SIG values with (a) serum phosphate, (b) creatinine, (c) albumin, and (d) chloride in AKI patients.

In our current study, we analyzed how the blood physicochemical parameters influenced the risk of mortality in critically ill patients with metabolic acidosis at 24 h, 72 h, 1 week, 1 month, and 3 months following the onset of AKI. The serum level of Cr and the values of SIDa, SIG, and AG were significantly higher among the survivors in the AKI group, compared with those of the nonsurvivors (Figure 1; Table 2). A higher SIDa value was associated with survival in the AKI group, which is consistent with the findings of a previous observational study of ICU patients [14]. The increased SIDa values among our AKI patients may have been the result of reductions in serum chloride that were not statistically significant (Figure 2(d)). Such differences might be explained by better chloride excretion among the survivors in the AKI group. This was consistent with a previous study where ICU survivors demonstrated a greater ability to progressively adjust their metabolic acid-base profile through chloride excretion, compared with that of their counterparts who did not survive [14]. Since survivors have lower chloride with higher creatinine level, it may also deduce that an earlier recognition of fluid status and appropriate fluid restriction policy have been given in these patients.

The patients in our study who survived AKI had higher serum creatinine levels and higher SIG values than those of the nonsurvivors. A positive correlation between SIG and Cr was also noted (Figure 2(b)) in the AKI patients. The correlation between a lower serum creatinine level and mortality may be explained by the dilution of serum creatinine resulting from volume overload or reduced muscle mass in the nonsurvivors. This positive correlation between serum creatinine and survival in our study is consistent with findings of previous studies [15–17]. Although more strong ions were observed under septic conditions [18], sepsis was not associated with a higher SIG value for same level of creatinine [12]. Therefore, a high SIG may be secondarily related to impaired renal function in patients with an increased level of creatinine as a result of decreased renal clearance of unmeasured anions, such as sulfate, rather than chloride or sepsis related anions [19, 20]. The SIG levels in the AKI patients in our study may also be influenced by the underlying disease condition and therapeutic measures. The significantly higher concentration of serum albumin, which contributed to higher SIG, might have reduced the alkalinizing effect of hypoalbuminemia and contributed to acidosis in the surviving AKI patients (Figure 2(c)). This finding is consistent with those of previous studies [8, 12]. Although the AG was higher among the AKI survivors, their albumin corrected AG was not significantly different from that of the nonsurvivors, which may have been the result of significant hypoalbuminemia among the nonsurvivors. With partially compensatory hyperchloremia, hypoalbuminemic metabolic alkalosis may have contributed to less acidotic conditions in the nonsurvivors. Therefore, severe malnutrition and the alkalinizing effect of hypoalbuminemia might have masked the presence of pathologic acids and delayed effective interventions in AKI patients who did not survive.

Multiple scoring systems, including the APS, APACHE II, and APACHE IV, have been used widely in clinical practice to predict outcome in ICU patients [21, 22]. We used the APS, APACHE II, and APACHE IV to evaluate prognoses and analyzed the correlation between these clinical variables and blood physicochemical parameters. Previous studies have shown that a higher SIG value is a better predictor of mortality than SBE, AG, and serum lactate [23–26]. However, the result of one previous study did not support such findings [27]. Our non-AKI acidotic patients had lower SIG values than the AKI patients, whereas other acid-base parameters were similar between the 2 groups. Higher SIG correlated with higher APACHE-II and -IV scores in the non-AKI group. However, in the AKI group, the APACHE-IV scores negatively correlated with the SIG value (*r* = −0.403, *P* < 0.05) and the level of serum albumin (*r* = −0.345, *P* < 0.05). We also found that the SIG value and the serum levels of creatinine and albumin decreased with increasing APACHE-IV scores, whereas the chloride levels increased with increasing APACHE-IV scores (Table 3). This finding indicates that higher Cr, SIG, and albumin and lower chloride levels are indicative of a better prognosis and survival in AKI patients. In the correlational analysis of the relationship between mortality and SIG, AG, and CAG over time, AG was associated with mortality at 1 and 3 months post-AKI (Table 4). However, SIG was associated with mortality at 24 h, 72 h, 1 week, 1 month, and 3 months post-AKI. Thus, SIG was a better predictor of overall mortality than AG and CAG in acidotic patients with AKI.

Our findings are subject to certain limitations. First, the size our cohort was relatively small and included only patients with metabolic acidosis at the time of ICU admission. Second, the underlying mechanism of AKI was not determined in our patients. Third, we did not perform an analysis of the serial physicochemical data because data on fluid, sodium, and chloride intake and output were not recorded before and after ICU admission. However, to the best of our knowledge, this is the first investigation of the prognostic value of blood physicochemical parameters as predictors of AKI survival in patients with metabolic acidosis at the time of ICU admission.

## 5. Conclusions

We performed an analysis of mortality based on Stewart acid-base parameters in critically ill metabolic acidosis patients with AKI. Given that the SIG value and the serum levels of creatinine, chloride, and albumin correlated with the APACHE-IV scores and mortality in the AKI patients, their fluid and nutritional statuses may play a vital role in their survival. Whether a high or low SIG value correlates with mortality in patients with metabolic acidosis and acute renal failure depends on the serum levels of creatinine, chloride, albumin, and phosphate. The SIG value may be a better predictor of both short- and long-term mortality from 24 h to 3 months post-AKI, whereas the AG value may predict mortality at 1 and 3 months only. Moreover, the SIG value is a better predictor of overall mortality than the AG and CAG values in AKI patients. Thus, the evaluation of blood physiochemical factors should be considered for monitoring and predicting the prognosis of critically ill patients with metabolic acidosis and AKI.

## Figures and Tables

**Table 1:** Characteristics of the ICU patients with metabolic acidosis.

	Nonacute kidney injury (*n *= 38)	Acute kidney injury (*n *= 40)	*P *
Age (y)	67.3 ± 10.4	72.5 ± 17.9	0.120
Sex (M : F)∗	18 : 20	22 : 18	0.5
WBC count	13152 ± 4364	17496 ± 10664	0.022
Hct	33.71 ± 5.52	32.28 ± 6.94	0.319
BUN	42.1 ± 18.6	91.7 ± 54.8	<0.0001
Cr	2.16 ± 0.91	5.73 ± 3.46	<0.0001
Glucose	213 ± 194	276.7 ± 257.0	0.219
Uric acid	7.39 ± 2.70	9.82 ± 3.56	0.001
Lactate	63.3 ± 42.2	67.3 ± 61.7	0.743
albumin	2.89 ± 0.57	2.95 ± 0.60	0.650
Bilirubin	2.02 ± 2.22	1.75 ± 2.58	0.623
Na (meq/L)	139.41 ± 4.8	142.03 ± 10.05	0.150
K (meq/L)	4.39 ± 0.77	5.04 ± 1.59	0.024
Cl (meq/L)	108.7 ± 6.4	104.4 ± 10.9	0.036
Ca (meq/L)	8.3 ± 0.5	7.8 ± 1.2	0.023
Mg (meq/L)	2.43 ± 0.48	2.81 ± 0.84	0.017
Phosphate (meq/L)	5.2 ± 1.4	7.2 ± 3.4	0.001
pH	7.219 ± 0.110	7.195 ± 0.152	0.417
PaCO_2_	31.96 ± 5.72	31.71 ± 11.14	0.903
PaO_2_	174.79 ± 92.57	121.63 ± 61.66	0.004
HCO_3_	14.73 ± 2.92	12.89 ± 4.66	0.041
SBE	−11.68 ± 3.88	−13.68 ± 6.31	0.098
APA II	24.3 ± 4.7	32.1 ± 7.2	<0.0001
APA IV	98 ± 17.7	112.5 ± 30.4	0.013
aSID	28.70 ± 4.94	34.75 ± 7.61	<0.0001
SIG	10.62 ± 3.20	17.15 ± 7.63	<0.0001
AG	15.63 ± 5.01	20.42 ± 9.51	0.007
CAG	11.65 ± 4.96	15.87 ± 9.21	0.015

*Chi-square test.

**Table 2:** Grouping of acute kidney injury patients by survival and mortality.

	Survival (*n* = 20)	Mortality (*n* = 20)	*P *
Age (y)	68 ± 18.4	77 ± 16.6	0.111
Na (meq/L)	141.15 ± 7.9	142.9 ± 11.9	0.589
K (meq/L)	4.9 ± 1.24	5.2 ± 1.9	0.146
Cl (meq/L)	101.9 ± 10.1	107 ± 11.2	0.139
BUN	105.7 ± 52.9	77.7 ± 54.4	0.107
Cr	7.1 ± 3.7	4.4 ± 2.56	0.011
Glu	287.3 ± 227	278.1 ± 285.2	0.911
Ca (meq/L)	7.97 ± 1.24	7.63 ± 1.21	0.392
Mg (meq/L)	2.69 ± 0.75	2.93 ± 0.93	0.364
P (meq/L)	7.35 ± 4.23	7.14 ± 2.49	0.849
Uric acid	9.26 ± 3.12	7.14 ± 2.49	0.322
Lactate	63.1 ± 70.04	71.4 ± 53.6	0.675
Albumin	3.28 ± 0.48	2.63 ± 0.54	<0.001
Bilirubin	1.5 ± 2.14	2.0 ± 2.99	0.547
pH	7.18 ± 0.18	7.21 ± 0.12	0.481
PaCO_2_	28.28 ± 10.48	35.14 ± 10.96	0.05
PaO_2_	124.3 ± 65.18	118.96 ± 59.51	0.788
HCO_3_	11.63 ± 5.33	14.15 ± 3.57	0.087
SBE	−14.99 ± 7.49	−12.37 ± 4.71	0.195
Hct	32.96 ± 7.98	31.59 ± 5.93	0.540
WBC count	18661.5 ± 11337.4	16331.5 ± 10106.4	0.497
APS	80.9 ± 21.5	111.1 ± 23.1	<0.001
APA. II	29.35 ± 7.68	34.8 ± 5.54	0.015
APA. IV	94.4 ± 24.4	130.6 ± 24.8	<0.001
a SID	37.24 ± 4.78	32.15 ± 8.89	0.032
SIG	20.85 ± 6.71	13.35 ± 6.18	0.001
AG	23.82 ± 11.31	17.15 ± 5.87	0.026
CAG	18.6 ± 11.24	13.13 ± 5.65	0.059

**Table 3:** Pearson correlation in acute kidney injury patients.

	SIG *r* (*P*)	AG* r* (*P*)	CAG *r *(*P*)	SBE *r* (*P*)	Lactate acid *r* (*P*)	Phosphate *r *(*P*)	Albumin *r* (*P*)
Age (y)	−0.045 (0.785)	−0.134 (0.409)	−0.099 (0.544)	0.119 (0.465)	−0.084 (0.606)	0.003 (0.986)	−0.059 (0.719)
Sex	−2.69 (0.093)	−0.317 (0.046)	−0.341 (0.031)	0.052 (0.750)	−0.183 (0.257)	−0.187 (0.247)	−0.144 (0.374)
WBC count	0.328 (0.039)	0.717 (<0.001)	0.641 (<0.001)	−0.476 (0.002)	0.504 (0.001)	0.035 (0.832)	0.002 (0.989)
Hct	0.128 (0.431)	0.208 (0.198)	0.244 (0.129)	−0.040 (0.807)	0.285 (0.074)	−0.142 (0.383)	0.015 (0.929)
BUN	0.574 (<0.001)	0.123 (0.449)	0.087 (0.593)	0.101 (0.537)	−0.472 (0.002)	0.447 (0.004)	0.377 (0.016)
Cr	0.526 (<0.001)	0.199 (0.219)	0.151 (0.353)	−0.027 (0.868)	−0.326 (0.04)	0.416 (0.008)	0.311 (0.051)
K	0.396 (0.011)	0.110 (0.5)	0.172 (0.288)	−0.009 (0.955)	−0.137 (0.4)	0.347 (0.028)	0.233 (0.148)
Cl	−0.152 (0.348)	−0.314 (0.048)	−0.252 (0.116)	0.069 (0.671)	−0.157 (0.333)	−0.183 (0.262)	−0.162 (0.318)
APA II	−0.013 (0.935)	0.193 (0.234)	0.199 (0.219)	−0.156 (0.338)	0.258 (0.108)	0.067 (0.682)	−0.072 (0.659)
APA IV	−0.403 (0.010)	−0.114 (0.485)	−0.047 (0.775)	−0.027 (0.868)	0.337 (0.034)	−0.254 (0.114)	−0.345 (0.029)
Phosphate	0.49 (<0.001)	0.261 (0.104)	0.120 (0.461)	−0.206 (0.201)	−0.122 (0.452)	1	0.281 (0.079)
Uric acid	0.146 (0.368)	0.124 (0.446)	0.107 (0.511)	−0.105 (0.520)	−0.022 (0.893)	0.423 (0.007)	0.124 (0.446)
Lactate	−0.046 (0.777)	0.645 (<0.001)	0.676 (<0.001)	−0.532 (<0.001)	1	−0.122 (0.452)	−0.060 (0.711)
albumin	0.547(<0.001)	0.392 (0.012)	0.258 (0.108)	−0.187 (0.247)	−0.060 (0.711)	0.281 (0.079)	1

**Table 4:** Pearson correlation of mortality with SIG, AG, and CAG.

	Nonselected (*n *= 78)			Acute renal failure (*n *= 40)		
	SIG	AG	CAG	SIG	AG	CAG
Day 1	−0.285∗	0.081	0.097	−0.227	−0.037	−0.017
Day 3	−0.222	−0.126	−0.125	−0.347∗	−0.230	−0.222
1 wk	−0.229	−0.087	−0.099	−0.362∗	−0.189	−0.196
1 mo	−0.292∗	−0.139	−0.122	−0.465∗∗	−0.346∗	−0.310
3 mos	−0.400∗∗	−0.113	−0.079	−0.512∗∗	−0.355∗	−0.301
Overall Mortality	−0.328∗	−0.417∗∗	−0.370∗∗	−0.512∗∗	−0.355∗	−0.301

*Correlation is significant at *P *< 0.05.

∗∗Correlation is significant at *P *< 0.01.
