# Corrigendum to “Metabolic Acidosis and Strong Ion Gap in Critically Ill Patients with Acute Kidney Injury”

**DOI:** 10.1155/2018/6380951

**Published:** 2018-06-24

**Authors:** Cai-Mei Zheng, Wen-Chih Liu, Jing-Quan Zheng, Min-Tser Liao, Wen-Ya Ma, Kuo-Chin Hung, Chien-Lin Lu, Chia-Chao Wu, Kuo-Cheng Lu

**Affiliations:** ^1^Division of Nephrology, Department of Internal Medicine, Shuang Ho Hospital, Taipei Medical University, Taipei, Taiwan; ^2^Department of Internal Medicine, School of Medicine, College of Medicine, Taipei Medical University, Taipei, Taiwan; ^3^Graduate Institute of Clinical Medicine, College of Medicine, Taipei Medical University, Taipei, Taiwan; ^4^Division of Nephrology, Department of Internal Medicine, Yonghe Cardinal Tien Hospital, New Taipei City, Taiwan; ^5^Department of Internal Medicine, Cardinal Tien Hospital, School of Medicine, Fu-Jen Catholic University, New Taipei, Taiwan; ^6^Department of Pediatrics, Taoyuan Armed Forces General Hospital, Taoyuan 325, Taiwan; ^7^Division of Nephrology, Department of Medicine, Shin Kong Wu Ho-Su Memorial Hospital, Taipei, Taiwan; ^8^Division of Nephrology, Department of Medicine, Tri-Service General Hospital, National Defense Medical Center, Taipei, Taiwan

In the article titled “Metabolic Acidosis and Strong Ion Gap in Critically Ill Patients with Acute Kidney Injury” [1], there was a missing affiliation for the first author. The revised authors' list and affiliations are shown above.
